# Factors associated with job and personal satisfaction in adult Brazilian intensivists

**DOI:** 10.5935/0103-507X.20160024

**Published:** 2016

**Authors:** Antonio Paulo Nassar Junior, Luciano César Pontes de Azevedo

**Affiliations:** 1 Discipline of Clinical Emergencies, Hospital das Clínicas, Faculdade de Medicina, Universidade de São Paulo - São Paulo (SP), Brazil.; 2 A.C. Camargo Cancer Center - São Paulo (SP), Brazil.; 3 Hospital Sírio-Libanês - São Paulo (SP), Brazil.

**Keywords:** Job satisfaction, Personal satisfaction, Physicians, Intensive care units, Questionnaires

## Abstract

**Objective:**

To evaluate job and personal satisfaction rates in physicians who work in adult intensive care units and to identify the factors associated with satisfaction.

**Methods:**

A cross-sectional study performed with physicians who participated in two intensive medicine online discussion groups. A questionnaire designed to assess the physician's sociodemographic profile and job was available for both groups for 3 months. At the end of the questionnaire, the participants addressed their degrees of job and personal satisfaction using a Likert scale in which 1 represented "very dissatisfied" and 5 represented "very satisfied". The association between sociodemographic and job characteristics with job and personal satisfaction was evaluated. Variables independently associated with satisfaction were identified using a logistic regression model.

**Results:**

The questionnaire was answered by 250 physicians, of which 137 (54.8%) declared they were satisfied with their jobs and 34 (13.5%) were very satisfied. None of the evaluated characteristics were independently associated with job satisfaction. Regarding personal satisfaction, 136 (54.4%) physicians reported being satisfied, and 48 (19.9%) reported being very satisfied. Job satisfaction (OR = 7.21; 95%CI 3.21 - 16.20) and working in a university hospital (OR = 3.24; 95%CI 1.29 - 8.15) were factors independently associated with the personal satisfaction of the participants.

**Conclusion:**

The participant physicians reported job and personal satisfaction with their work in intensive care. Job satisfaction and working in a university hospital were independently associated with greater personal satisfaction.

## Abstract (pt)

**Objetivo:**

Avaliar as taxas de satisfação profissional e pessoal, bem como os fatores associados a eles em médicos que atuam em unidades de terapia intensiva adulto.

**Métodos:**

Estudo transversal realizado com médicos que participavam de dois grupos on-line de discussão em Medicina Intensiva. Um questionário contendo perguntas referentes ao perfil sociodemográfico e à atuação profissional do médico foi disponibilizado nos dois grupos por um período de 3 meses. Ao final do questionário, os participantes respondiam qual era seu grau de satisfação profissional e pessoal, usando-se uma escala Likert, em que 1 indicava "muito insatisfeito" e 5, "muito satisfeito". Avaliou-se a associação de características sociodemográficas e profissionais com a satisfação profissional e pessoal. As variáveis independentemente associadas à satisfação foram identificadas por um modelo de regressão logística.

**Resultados:**

Responderam o questionário 250 médicos; 137 (54,8%) declararam estar satisfeitos profissionalmente e 34 (13,5%) muito satisfeitos profissionalmente. Nenhuma das características avaliadas foi independentemente associada à satisfação profissional. Do ponto de vista pessoal, 136 (54,4%) médicos disseram estar satisfeitos e 48 (19,9%) muito satisfeitos. Satisfação profissional (OR = 7,21; IC95% 3,21 - 16,20) e trabalhar em hospital universitário (OR = 3,24; IC95% 1,29 - 8,15) foram fatores independentemente associados à satisfação pessoal dos participantes.

**Conclusão:**

Os médicos participantes estavam satisfeitos do ponto de vista profissional e pessoal sobre sua atuação em medicina intensiva. Satisfação profissional e trabalhar em um hospital universitário foram independentemente associados à maior satisfação pessoal.

## INTRODUCTION

Intensive care units (ICUs) are environments with abundant work^(1)^ and conflict-related^(2)^ emotional stress. Professionals who work in ICUs exhibit a high prevalence of *burnout*^(3)^ and other psychological symptoms,^(4,5)^ which are associated with job dissatisfaction.^(6)^ Conversely, the practice of intensive care medicine can be rewarding, and the feeling of job satisfaction can overcome the effects of the stressful conditions.^(7)^

The physicians' job and personal satisfaction are important because they are associated with patient satisfaction^(8)^ and greater productivity.^(9)^ However, although the satisfaction of patients and family members is commonly evaluated, few studies have specifically assessed physician satisfaction.^(10)^ Although studies in Brazil have evaluated job satisfaction among physicians in general,^(11,12)^ no studies have specifically addressed job satisfaction in a population of adult intensivists.

Thus, the present study evaluated the job and personal satisfaction rates of physicians who worked in adult intensive care units and identified the factors associated with satisfaction.

## METHODS

A cross-sectional study was performed with physicians who participated in two intensive care medicine online discussion groups. A questionnaire prepared in the REDCap^(13)^ tool was sent via e-mail to the participants of both groups. The study was approved by the Ethics and Research Committee of the *Hospital das Clínicas* of the *Universidade de São Paulo* under registration number 13.973/2015. The participants were required to complete the Informed Consent Form prior to answering the questionnaire.

The questionnaire contained questions regarding sociodemographics (age, children, and region of work in Brazil), intensive care medicine training (residence and specialist title), the intensive care medicine practice (time, weekly workload, ICU type (i.e., open, in which most decisions are made by the physician who requested the bed in the ICU, or closed, in which most decisions are made by the intensivist physician)), the hospital in which they worked (public non-university, public university, or private), number of jobs, position held at the ICU (physician on duty, daily routine physician who visits hospitalized patients, daily physician on duty who works 6-hour shifts providing medical assistance to patients, or coordinator), mean monthly income and number of intensive medicine events attended in the last 5 years. The questionnaire also assessed job and personal satisfaction using the Likert scale (1 for very dissatisfied, 2 for dissatisfied, 3 for not satisfied nor dissatisfied, 4 for satisfied, and 5 for very satisfied). Participants could mark more than one answer regarding the hospital in which they worked and their position if they had more than one job; for example, a participant could be a "physician on duty" in a "private hospital" and a "coordinator" in a "public hospital". Thus, the participant would mark "physician on duty" and "coordinator" when asked about their position in the ICU and "private hospital" and "public hospital" when asked about the type of hospital in which they worked. The questionnaire was available to the group for 3 months (September to November, 2015). Reminders about the questionnaire were sent via e-mail to potential participants every 3 weeks.

### Statistical analysis

Categorical variables are presented as absolute numbers and percentages and were compared using the Chi-square or Fisher's text as suitable. Continuous variables are presented as medians and interquartile ranges and were compared using the Mann-Whitney test. The correlation between personal satisfaction and job satisfaction was evaluated using Spearman's correlation coefficient (ρ).

For analysis purposes, professionals who marked being "satisfied" or "very satisfied" were considered satisfied. Participants who marked the remaining options were considered "dissatisfied". The same definitions were used to evaluate personal satisfaction. Regarding the position held at the ICU, participants who reported working as coordinators and/or daily physicians (routine or on duty) were combined in one variable due to the hypothesis that intensivists who dedicated more time to their unit would exhibit a higher degree of satisfaction.

All variables that exhibited p < 0.2 in the univariate analysis were inserted into the two following binary logistic regression models (enter type): the first to evaluate factors independently associated with job satisfaction and the second to evaluate factors associated with personal satisfaction. The results of the logistic regression are presented as odds ratios (ORs) and 95% confidence intervals (95%CIs). Variables with p < 0.05 were considered significant. All analyses were performed using *Statistical Package for Social Science* (SPSS), version 21.0 (IBM, Armonk, NY, USA).

## RESULTS

In total, 250 physicians responded to the questionnaire. One participant responded to the questionnaire but did not complete the Informed Consent Form; his/her answers were not included in the study. The participants were predominantly male, young (median age of 37 years), had no children, had a specialist title, had worked in intensive care for less than 10 years and were located in the southeast region of Brazil (Table 1).

**Table 1:** Participant characteristics and factors associated with job satisfaction

**Participant characteristics**	**General** ** (N = 250)**	**Satisfied** ** (N = 171)**	**Dissatisfied** ** (N = 79)**	**p value**
Age (years)	37 (32 - 43)	37 (32 - 42)	37 (32 - 43)	0.68
Male	157 (62.8)	112 (65.5)	45 (57)	0.18
With children	116 (46.4)	82 (48.0)	34 (43.0)	0.52
Residence in intensive care medicine	127 (50.8)	86 (50.3)	41 (51.9)	0.88
Boarding certified in intensive care medicine	154 (61.6)	106 (62.0)	48 (60.8)	0.72
Total weekly workload (hours)	60 (48 - 72)	60 (45 - 70)	60 (50 - 74)	0.06
Time working in ICUs (years)	9 (4 - 15)	10 (4 - 16)	9 (5 - 15)	0.69
Weekly ICU workload (hours)	44 (30 - 60)	44 (30 - 60)	42 (30 - 60)	0.91
Number of ICU jobs	2 (1 - 2)	2 (1 - 2)	2 (1 - 2)	0.83
Practices other specialty	113 (45.2)	75 (43.9)	38 (48.1)	0.42
Mean monthly income (in thousand BRL)	20 (15 - 28)	20 (15 - 28)	18 (15 - 30)	0.30
Proportion of income from working in ICUs (%)	90 (60 - 100)	95 (60 - 100)	85 (60 - 100)	0.47
Works in general ICU	227 (90.8)	156 (91.2)	71 (89.9)	0.73
Works in closed ICU	144 (57.6)	96 (56.1)	48 (60.8)	0.45
Type of hospital in which they work				
Public	57 (22.6)	40 (23.4)	17 (21.5)	0.74
University	79 (31.3)	52 (30.4)	27 (34.2)	0.55
Private	185 (73.4)	126 (73.7)	58 (73.4)	0.96
Coordinator and/or daily physician	157 (62.8)	116 (67.8)	41 (51.9)	0.02
Number of patients under their responsibility in one shift	10 (8 - 10)	10 (8 - 10)	10 (10 - 10)	0.01
Number of intensive care medicine events attended in the last 5 years	5.5 (4 - 10)	6 (4 - 10)	5 (3 - 10)	0.05
Region in which they work in ICUs				0.76
North	2 (0.8)	2 (1.2)	0 (0)	
Northeast	34 (13.5)	21 (12.3)	13 (16.5)	
Midwest	5 (2.0)	3 (1.8)	2 (2.5)	
Southeast	192 (76.2)	132 (77.2)	59 (74.7)	
South	17 (6.7)	12 (7.0)	5 (6.3)	

ICU - intensive care unit. Results expressed as number, percentages and medians.

In total, 137 (54.8%) and 34 (13.5%) physicians declared being satisfied and very satisfied with their jobs, respectively. Four (1.6%) physicians declared being very dissatisfied and 37 (14.7%) declared being dissatisfied with their careers. A total of 38 physicians (15.1%) reported they were neither satisfied nor dissatisfied with their jobs.

The factors associated with greater job satisfaction in the univariate analysis were male gender, lower weekly workload, work as a coordinator and/or daily physician in the ICU and attended a greater number of scientific events over the last 5 years. Being responsible for a larger number of patients during the work shift was associated with lower job satisfaction (Table 1). However, none of these factors was independently associated with job satisfaction in the multivariate analysis with logistic regression (Table 2).

**Table 2:** Multivariate analysis of factors associated with job satisfaction

**Variable**	**OR (95%CI)**	**p value**
Male	1.23 (0.60 - 2.51)	0.57
Weekly workload (hours)	0.99 (0.98 - 1.01)	0.48
Work as coordinator and/or daily physician	1.63 (0.82 - 3.23)	0.16
Number of participants under their responsibility in one shift	0.88 (0.76 - 1.02)	0.07
Number of ICU events attended in the last 5 years	1.02 (0.96 - 1.09)	0.49

OR - odds ratio; 95%CI - 95% confidence interval; ICU - intensive care unit.

Regarding personal satisfaction, 136 (54.4%) physicians reported being satisfied, and 48 (19.9%) reported being very satisfied. In total, 27 (10.7%) physicians reported that they were neither satisfied nor dissatisfied, 35 (13.9%) reported that they were dissatisfied, and 4 (1.6%) reported that they were very dissatisfied with their personal life.

**Figure 1:**
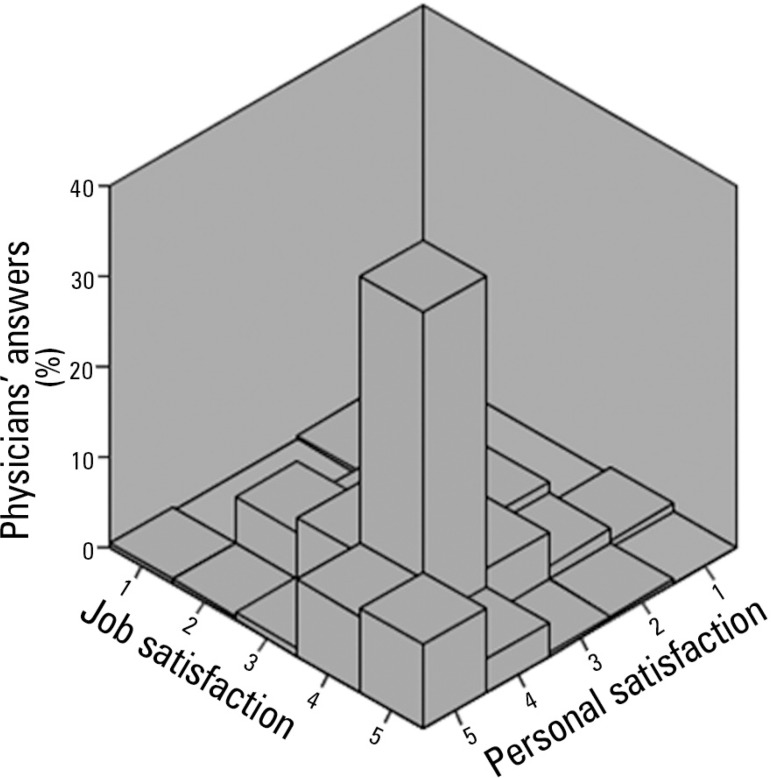
Job and personal satisfaction of the participating physicians. 1 - Very dissatisfied; 2 - Dissatisfied; 3 - Not satisfied nor dissatisfied; 4 - Satisfied; 5 - Very satisfied.

Job satisfaction was positively associated with personal satisfaction (p = 0.54; p < 0.01). The proportion and agreement between the answers regarding job and personal satisfaction are shown in figure 1.

Age, being board certified, working fewer hours per week, working longer in the ICU, participating in a greater number of scientific events over the last 5 years, not being a physician on duty, being a coordinator and/or a daily physician, working in a university hospital and having a lower number of patients under their responsibility during their shift were factors associated with greater personal satisfaction (Table 3). In the multivariate analysis, job satisfaction (OR = 7.21; 95%CI 3.21 - 16.20) and working in a university hospital (OR = 3.24; 95%CI 1.29 - 8.15) were factors that were independently associated with the personal satisfaction of the participants (Table 4).

**Table 3:** Factors associated with the personal satisfaction of participants

**Participant characteristics**	**Satisfied** ** (N = 184)**	**Dissatisfied** ** (N = 66)**	**p value**
Age (years)	37 (32 - 44)	36 (32 - 41)	0.07
Male	118 (63.8)	39 (59.1)	0.44
With children	84 (45.7)	32 (48.5)	0.72
Residence in intensive care medicine	91 (49.5)	36 (54.5)	0.53
Board certified in intensive care medicine	119 (64.7)	35 (53.0)	0.07
Total weekly workload	60 (44 - 70)	60 (52 - 80)	0.01
Time working in ICUs (years)	10 (5 - 17)	8 (4 - 13)	0.01
Weekly ICU workload (hours)	42 (30 - 60)	48 (30 - 60)	0.32
Number of ICU jobs	2 (1 - 2)	2 (1 - 2)	0.42
Practices other specialty	82 (44.6)	31 (47.0)	0.69
Mean monthly income (in thousand BRL)	20 (15 - 28)	19 (15 - 24.75)	0.29
Proportion of income from working in ICUs (%)	90 (60 - 100)	90 (60 - 100)	0.68
Works in general ICU	169 (91.8)	58 (87.9)	0.34
Works in closed ICU	105 (57.1)	39 (59.1)	0.71
Type of hospital in which they work			
Public	39 (21.1)	18 (27.3)	0.30
University	64 (34.6)	15 (22.7)	0.07
Private	134 (72.8)	50 (75.8)	0.64
Coordinator and/or daily physician	122 (66.3)	35 (53.0)	0.06
Number of intensive care medicine events attended in the last 5 years	6 (4 - 10)	5 (3 - 10)	0.16
Region in which they work in ICUs			0.53
North	2 (1.1)	0 (0.0)	
Northeast	25 (13.5)	9 (13.6)	
Midwest	5 (2.7)	0 (0.0)	
Southeast	141 (76.2)	51 (77.3)	
South	11 (5.9)	6 (9.1)	
Job satisfaction	148 (80.4)	23 (34.8)	< 0.01

ICU - intensive care unit. Results expressed as number, percentages and medians.

**Table 4:** Multivariate analysis of factors associated with personal satisfaction

**Variable**	**OR (95%CI)**	**p value**
Job satisfaction	7.21 (3.21 - 16.20)	< 0.01
Age	0.93 (0.79 - 1.09)	0.37
Board certification in ICU	1.12 (0.93 - 1.34)	0.23
Weekly workload	0.99 (0.97 - 1.01)	0.19
Time working in ICUs	1.12 (0.93 - 1.34)	0.23
Number of ICU events attended in the last 5 years	0.99 (0.93 - 1.05)	0.75
Coordinator and/or daily physician	1.02 (0.43 - 2.41)	0.96
Working in an university hospital	3.24 (1.29 - 8.15)	0.01
Number of patients under their responsibility in one shift	0.92 (0.77 - 1.08)	0.30

OR - odds ratio; 95%CI - 95% confidence interval; ICU - intensive care unit.

## DISCUSSION

Our study was the first to evaluate the job and personal satisfaction of physicians working in adult ICUs in Brazil. Most participant physicians (67.9%) were satisfied with their professional path, and most (73.4%) were also satisfied with their personal life. Job satisfaction and practicing intensive care medicine in a university hospital were associated with greater personal satisfaction.

A North American study evaluated satisfaction among several medical specialties. The practice of intensive care medicine as a subspecialty of internal medicine was only the 20^th^ of 42 specialties in terms of satisfaction scores. When intensive care medicine was considered a subspecialty of pulmonology, the results were even worse (41^st)^.^(10)^ Previous studies that evaluated Brazilian physicians with other specialties found satisfaction rates similar to those of the present study.^(11,12)^ However, another Brazilian study showed that pediatric intensivists had greater burnout rates than general pediatricians.^(14)^ It is well known that burnout is closely related to job satisfaction.^(6)^

The high satisfaction rate in our study differed from that of a previous survey performed with pediatric intensivists, which found a dissatisfaction rate of 63%.^(15)^ Although the surveys were performed with different specialties, it was interesting that in the present study, the proportion of physicians who were exclusively dedicated to intensive care medicine was greater (54.8% *versus* 40%), as also was the proportion of board certified physicians (61.5% *versus* 33%). A previous study showed lower burnout symptoms in physicians who specialized in intensive care medicine compared to those who worked in the ICU but had a degree in other specialties.^(16)^ Thus, having the possibility of exercising their chosen specialty seemed to be a factor associated with job satisfaction.^(17)^ As previously demonstrated, job and personal satisfaction were positively correlated.

Working in an academic environment and having contact with students and residents was a factor that was independently associated with greater personal satisfaction in the present study. This finding is similar to the North American study that evaluated satisfaction in different medical specialties.^(10)^ A survey performed with Brazilian physicians suggested that having a doctorate was associated with greater professional satisfaction.^(18)^

Several studies have shown an association between higher salaries and job satisfaction.^(10,12,15)^ In the present study, we did not find any association between income and personal or job satisfaction. However, the mean monthly income was higher than the income in the two previous Brazilian studies.^(12,15)^ One explanation for the lack of association between income and satisfaction found in our study is that there was no association with emotional well-being after a certain level of income was obtained.^(19)^

Other factors commonly associated with the job satisfaction of physicians are workload^(10,20)^ and autonomy.^(11,17)^ In the present study, neither factor was associated with job or personal satisfaction. Although the number of weekly working hours of the participants was high (median of 60 hours) compared with the medians in the North American^(10)^ and European studies,^(3,21)^ it was lower than that of other Brazilian studies with adult^(16)^ and pediatric intensivists.^(14)^ In the study of adult intensivists,^(16)^ most participants had no specific education for the specialty; thus, working long hours in an area that was not chosen might be related to their dissatisfaction. The study with pediatric intensivists did not show data regarding a residency or specialist title in intensive care medicine.^(14)^

Regarding autonomy, the units that were more closed (i.e., those in which the intensivist has greater control over patient conduct) were associated with greater satisfaction.^(22)^ The study of intensivists conducted in Salvador (BA) showed that greater control over work was associated with lower burnout rates.^(23)^ The present study did not show any association between ICU type (open *versus* closed) and job satisfaction. Some hypotheses can be developed to explain these differences. First, there might not be a relationship between ICU type and satisfaction. Second, the definition of open and closed ICU may not be clear for the Brazilian intensivist, or "control" or "autonomy" may be perceived differently in different occasions regardless of the ICU type.

Our study has several limitations. First, the participants represent a convenience sample with some bias regarding Brazilian physicians that work in ICUs. This limitation is noticeable when we examine the proportion of participants from the Southeast region (76.2%) in the survey compared with the number of ICUs in this region (52.7%) according to the Census of the Brazilian Association of Intensive Care Medicine (Censo da Associação de Medicina Intensiva Brasileira - AMIB).^(24)^ Second, the study may have a small power to detect differences. For example, with a larger sample, the number of patients under the responsibility of a physician during a shift may be associated with lower job and personal satisfaction. The use of ordinal regression instead of binary logistic regression would have given more power to the study, but the original study protocol expected that a binary logistic regression would be performed to identify factors independently associated with job and personal satisfaction. In any case, in an exploratory analysis, we performed a multivariate analysis using *post hoc* ordinal regression; however, this approach did not produce significantly different results from those found using binary logistic regression. Third, this study was conducted by sending the questionnaire to intensive care medicine online discussion groups; thus, these physicians may have been more involved and satisfied with the practice of the specialty because they read and discuss scientific texts. Therefore, the high satisfaction levels found may not be representative of the Brazilian population of physicians that work in intensive care. Fourth, the satisfaction assessment was based on a questionnaire that had not been previously evaluated. However, this approach is similar to the approach used in other studies.^(10)^ Additionally, because the questions were not validated and the participants were asked to consider a specific period (e.g., the last year) to quantify their job and personal satisfaction, an isolated event (e.g., a good result in a difficult case) that occurred in a moment close to the period in which the questionnaire was answered could have influenced the participants' answers.

## CONCLUSION

Most of the doctors that worked in intensive care units were satisfied with the professional and personal paths in their lives. Job and personal satisfaction were positively correlated. Physicians who worked in the intensive care units of university hospitals were more satisfied from the personal point of view. Future studies with more representative samples of the population of physicians that work in intensive care units should be performed to obtain a better evaluation of the satisfaction of these professionals and the factors that influence their satisfaction.
